# Methodological Basis for Reliable Evaluation of Air Void Structure Parameters Using the 2D Method

**DOI:** 10.3390/ma18092095

**Published:** 2025-05-02

**Authors:** Jerzy Wawrzeńczyk, Henryk Kowalczyk

**Affiliations:** Faculty of Civil Engineering and Architecture, Kielce University of Technology, Al. Tysiąclecia Państwa Polskiego 7, 25-314 Kielce, Poland; zmsjw@tu.kielce.pl

**Keywords:** Monte Carlo method, concrete, spacing factor, air void structure, 2D surface method

## Abstract

Frost resistance of pavement concrete is closely related to air void structure. Traditionally, such a structure is assessed by measuring chord lengths according to the guidelines provided in the PN-EN 480-11 standard. In recent years, increased attention has been given to analyzing pore diameters (2D) on the surface of concrete samples. The measurement procedure employed in the surface method should enable accurate identification of small pores formed by modern air-entraining admixtures. Researchers suggest only pores under 300 µm significantly impact frost resistance, raising the question of whether pores over 1000 µm should be considered in measurements. This study attempts to define the measurement frame parameters required to obtain satisfactory results. Additionally, a comparative analysis of air void structure parameters from 2D and 1D methods was conducted. Geometrical models of air voids distributed within cement paste using the Monte Carlo method based on air void structure data derived from real concrete were created. Analysis of these models demonstrated good agreement between the 2D and 1D results. It was concluded that satisfactory results require the analysis of either three measurement frames of 50 × 50 mm or four frames of 40 × 40 mm, with a resolution of at least 3 µm/px.

## 1. Introduction

The analysis of air voids in concrete is a commonly used method for estimating the potential frost resistance of concrete. The primary parameter for evaluating the quality of air-entrained concrete is the spacing factor *L*. A model for calculating this indicator was developed by Powers in the 1940s [1]. The calculation method for the *L* parameter was first adopted in the American standard ASTM C457 [2] and later in the European standard EN 480-11 [3].

The microscopic method involves counting the lengths of chords formed when an air void is intersected by a traverse line. Several criticisms have been raised regarding the method of calculating this parameter. Primarily, it has been noted that the calculation is based on the average chord length without considering the actual distribution of pore sizes [4,5]. Attiogbe [6] observed that large pores significantly influence the average chord length; therefore, the pore (chord) size included in the analysis should be limited to 2 mm.

Snyder et al. [5] conducted a study on the impact of the number of chords on air void structure parameters. Their analysis demonstrated that when the air content is low or when there are many large pores, the chord count and length may be insufficient for the precise estimation of parameters. They concluded that at least 1000 measured chords are necessary to obtain reliable results.

In recent years, there has been a growing interest in the application of automated 2D image analysis [7,8,9,10,11]. Unlike the traverse method, the 2D method analyzes a fragment of the polished surface. However, a precise methodology for conducting studies using this approach has not yet been defined. Various methods for concrete sample preparation, contrast enhancement, image acquisition, measurement procedures, and data analysis of pore distribution have been described by Song [7], Arnesh [12], Fonseca and Scherer [11], as well as in the authors’ previous work [10].

Concrete is a heterogeneous material composed of cement paste, air voids, and aggregate, making it necessary to analyze a large polished surface area. According to the C457 standard, the dimensions of measurement frames should depend on the maximum aggregate particle size. The measurement window should also be sufficiently large to account for pores exceeding 1000 µm. In the case of small measurement windows, such large pores can significantly influence the results and may lead to overestimation, typically increasing the *L* parameter [13,14,15]. It is also recognized that, from a frost resistance perspective, pores smaller than 300 µm (micropore content *A*_300_) are the most relevant, while pores larger than 350 µm have no significant impact on frost resistance [16]. This raises the question of whether pores larger than 350 µm should be included in the calculations.

The analysis of air-entraining admixtures has shown that modern admixtures generate significantly finer pores (d < 10 µm) compared to older admixtures [17]. The resolution of measurement frames should allow for the detection of such fine pores. The use of large-sized measurement frames with high resolution (to account for the need to measure pores smaller than 10 µm) leads to an increase in file size. Given technical and hardware limitations, working with files of large volume may be challenging or even infeasible. Table 1 presents the parameters of measurement frames used by various researchers.

This creates a need for an analysis of the impact of measurement frame size and resolution on the obtained results. Conducting such studies on concrete samples is costly, time-consuming, and requires significant labor input. A potential solution to this issue is the use of geometric models of concrete surfaces [21]. Based on real concrete data, including air void shape, size, and distribution, a dataset necessary for model generation can be created. This approach enables the preparation of a large number of models for statistical analysis within a relatively short time. Snyder [21] stated that simulating a large number of concrete models could allow for the evaluation of the effectiveness of the aforementioned 2D surface method. According to him, the Monte Carlo method can be used to simulate data for geometric concrete models. In his study [22], he also conducted a computational experiment to calculate the spacing factor using the formulas proposed by Powers [23], Philleo [24], Pleau and Pigeon [25], and Attiogbe [6]. His mathematical model allowed for tracking pore sizes and distributions based on their size distribution.

The concept of modeling the structure of various materials and objects to simulate their properties emerged as early as the early 1980s [26,27]. Initially, only numerical models were developed. Examples include models related to flexural strength and crack formation in concrete.

Zaitsev and Wittmann [28] presented a visualization of cracking in a concrete model containing aggregate. By simplifying the actual concrete structure, the authors proposed four levels characterizing geometric concrete models: (i) macro—where only large aggregate particles are present in the cement paste, (ii) meso—where both large and fine aggregate particles are included, (iii) micro—where large air voids also appear, and (iv) nano—where the model structure consists of both fine and coarse aggregate, as well as small and large pores.

An additional modeling level was described by Unger and Eckardt [29]. The authors introduced a hybrid (multi-scale) approach, where the model structure is represented at the macro scale, while critical areas (such as cracks) are analyzed at the meso scale. A similar approach has been employed by other researchers [30,31,32].

Wiggers and Moftah [33] utilized the Monte Carlo method to generate data based on aggregate size distribution. Increasingly precise 3D models have been developed by researchers such as Stefan et al. [34], Leite et al. [35], and Lilliu and van Mier [36].

The fundamental model of air voids was introduced by Powers [1], who assumed that pores are arranged at the corners of a cube. Wawrzeńczyk and Kozak [37] utilized geometric models of air voids in concrete to analyze air void structure parameters. Their study was conducted using the 2D surface method, the 1D traverse method, and the assessment of the protected paste surface based on Philleo’s factor [24]. Authors employed an idealized concrete structure that accounted for pore size distribution, aggregate distribution, and the influence of aggregate on pore arrangement. The use of models for determining the protected paste surface was also explored in the work of Yu Song et al. [38].

In recent years, non-destructive concrete analysis using computed tomography (CT) with the 3D method has been developing rapidly. This technique allows for the measurement of the shape and size of pores within the analyzed concrete volume. It enables the determination of pore size distribution directly in 3D space without the need for sample preparation. The first use of CT for analyzing air voids in concrete was recorded in 2001 [39]. Weise demonstrated an analysis of a cylinder with a diameter of 8mm. Although the author did not measure the number of pores, he demonstrated the ability to distinguish pores as small as 0.1 µm. Cnudde et al. [40] concluded that CT scan results significantly complement existing knowledge. However, they noted that measurements are highly limited by the maximum sample size and relatively low resolution.

Kim et al. [19] analyzed flat cross-sections obtained from computed tomography. The study focused on determining the *L* factor and estimating the distribution of pore diameters in 3D space. The authors reconstructed a concrete sample model and identified air voids. Subsequently, two-dimensional cross-sections of the model were extracted, on which measurements and calculations were performed using the traverse method. The study also led to the development of pore size distributions based on diameters recorded in the sphere model.

Jóźwiak-Niedźwiedzka et al. [41] compared the air content in concrete samples determined using the traverse method under a microscope with computed tomography reconstructions. They observed that the difference between the obtained results was less than one percentage point. The authors also analyzed the micropore content *A*_300_. However, in this case, greater discrepancies were found, reaching up to three percentage points. This divergence was attributed to the relatively large sample size used in CT analysis (10 × 10 cm), which limited the achievable resolution.

Sang-Yeop Chung et al. [42] conducted air void size measurements using 1D, 2D, and 3D methods. Their findings indicated that the distributions obtained using the 1D and 2D methods were almost identical. Additionally, both methods showed good agreement with measurements in CT. However, due to significant hardware limitations, the analysis was restricted to small concrete samples. In many cases, low resolution raised serious concerns regarding the quality of the obtained analysis results [43].

This study presents an attempt to determine the impact of resolution and measurement frame size to enable the detection of pores smaller than 10 µm while ensuring that the analysis yields satisfactory results. A comparative analysis of air void structure parameters calculated using the 1D and 2D methods was conducted.

## 2. Materials and Methods

The primary objective of the research was to determine guidelines for the size and resolution of measurement frames to obtain reliable computational results for air void structure parameters. A comparative analysis of air void structure calculations using 1D and 2D methods was also conducted. Producing concrete with a specified air content and air void distribution is challenging; therefore, an alternative approach was adopted. This approach involves generating geometric models representing air voids distributed within the cement paste. The foundation of this method was based on air void structure studies conducted over the past five years on real pavement concretes at the Faculty of Civil Engineering and Architecture of the Kielce University of Technology. The analysis and research findings were presented in a previous study by the authors [10,16]. These concretes had a water-to-cement ratio (W/C) in the range of 0.37–0.39. The air void structure parameters using the 1D method were determined following the guidelines of the PN-EN 480-11 standard. A general characterization of the concretes is presented in Table 2.

**Figure 1:**
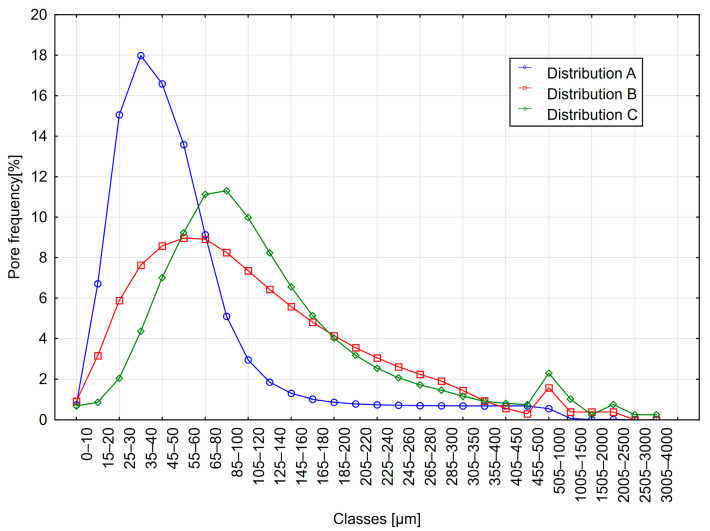
Pore size distributions of three selected concretes.

Three concretes with varying air void structures were selected for analysis. Their air void structure parameters were determined using the 2D method. Measurement frames with dimensions of 82.5 × 61.9 mm and a resolution of 6.75 µm/px were applied to measure the equivalent diameters of air voids. Subsequently, lognormal distributions describing the air void distribution were developed (Figure 1). These distributions served as the basis for generating geometric models of the concrete surface. Data for the models were generated in MATLAB R2023a, The MathWorks Inc., Natick, MA, USA [44], and images were created using the NIS-Elements AR 4.6 software, Nikon, Tokyo, Japan [45]. It was assumed that the images would contain only air voids and cement paste, similar to Powers’ model [1]. Aggregate grains were excluded from the analysis. Additionally, it was assumed that air voids could merge and cluster, as observed in real concretes.

**Figure 2:**
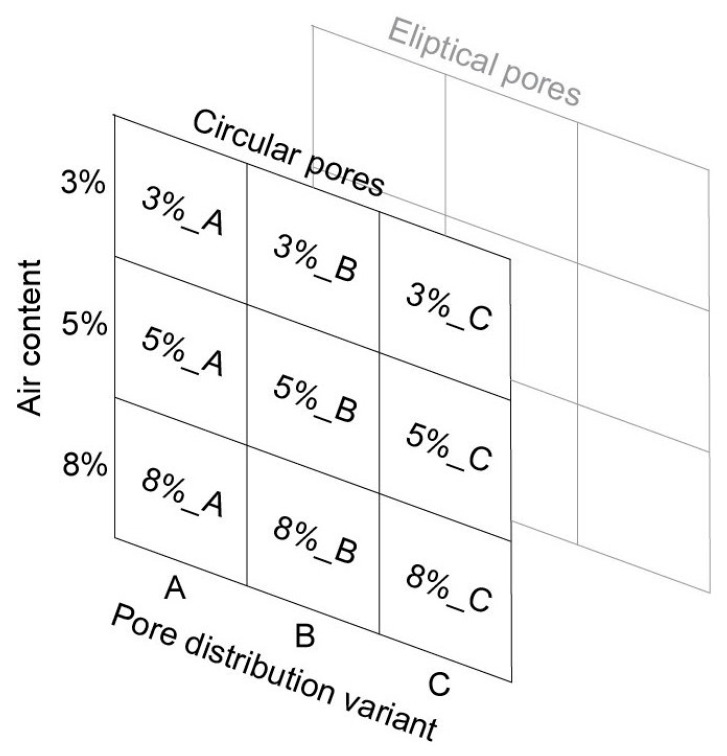
Diagram of the variants of generated digital images.

The research program considered four variables: (i) air void distribution type: A, B, C; (ii) air content: 3%, 5%, 8%; (iii) air void shape: circular, elliptical; and (iv) the rotation angle of the ellipse’s major chord: 0–180°. The schematic representation of the adopted research plan is shown in Figure 2. As a result, 18 geometric models of the concrete surface were generated, each with dimensions of 40,000 × 25,000 px and a resolution of 2 µm/px (80 × 50 mm).

### Methodology for Preparing Geometric Models of Air Voids in Concrete

**Figure 3:**
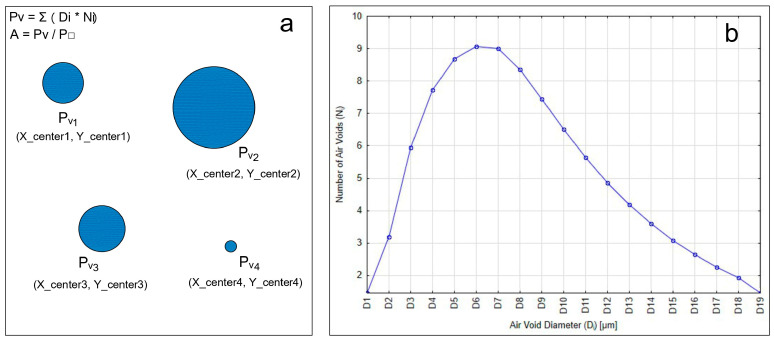
Determination of air void number and size (**a**) and distribution of air voids (**b**).

The first parameter necessary for generating the models was the planned air content (3%, 5%, 8%). The number and size of air voids were determined based on the assumption that the ratio of the total area of all air voids in the image (ΣPV) to the total image area (P□) equals the planned air content A (Figure 3). For calculations, the average pore diameter within each size class was used. Subsequently, the coordinates of the center of each air void (*x*_center, *y*_center) were determined.

**Figure 4:**
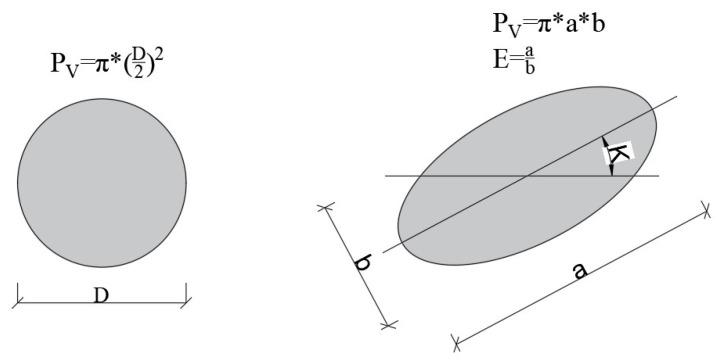
Area and dimensions of an ellipse and a circle.

**Figure 5:**
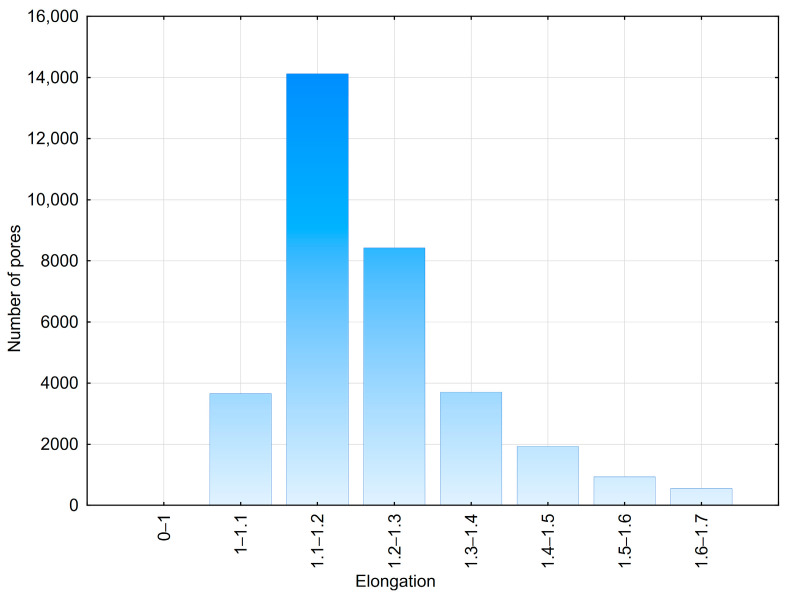
Elongation of air voids determined using 2D measurements.

The 2D analysis of pavement concretes revealed a diverse range of air void shapes. Therefore, it was decided to generate images containing both circular and elliptical air voids. Elliptical air voids are characterized by the elongation ratio E, which describes the ratio of the major axis to the minor axis of the ellipse (Figure 4). The values of this parameter were determined based on measurements conducted on real concretes (Figure 5).

For elliptical air voids, the elongation ratio E was set within the range of 1.0 to 1.4, with 1.0 indicating a perfectly round object. It was assumed that elliptical and circular air voids have the same surface area and are located in the same positions. The dimensions of ellipses were calculated from the diameters of circular air voids using the formulas presented in Figure 4. Additionally, a random rotation angle of the ellipse’s major axis was assigned within the range of 0–180°.

The specified parameters for individual air voids were determined using the Monte Carlo method [44]. This method relies on an algorithm known as the Mersenne Twister, a pseudorandom number generator commonly used in Monte Carlo simulations. The period length of this generator is defined by the 24th Mersenne prime number, which equals 2^19937^ − 1. The generated parameters were stored in an Excel file.

**Figure 6:**
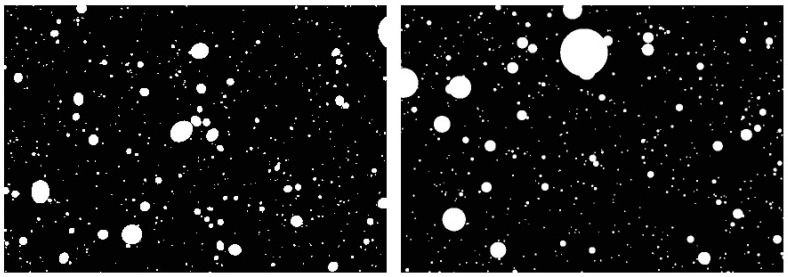
Examples of binary images with circular pores (**right**) and elliptical pores (**left**).

The next step involved generating digital images with distributed circular and elliptical air voids using NIS-Elements software. Initially, black images with dimensions of 40,000 × 25,000 px and a resolution of 2 µm/px (80 × 50 mm) were created. Generating larger images was not possible due to limitations of NIS-Elements. Handling files larger than 200 MB is considerably challenging and often impractical. A custom macro was designed to import data from an Excel file, capturing the position, dimensions, and shape of the air voids. Based on this data, Regions of Interest (ROI) were created to outline the shapes on the black images. The next step was converting the ROI layer into a binary layer. Example images featuring circular and elliptical voids are shown in Figure 6.

## 3. Results

Measurements were conducted on the generated images using both 2D and 1D methods. Chord length measurements and calculations of air void structure parameters by the linear traverse method were performed following the guidelines of the PN-EN 480-11 standard, with a traverse line spacing of 30 mm. The air content is calculated based on the total length of the chords *T_a_* and the total length of the measurement line *T_tot_* (Equation (1)).

(1) $$A=\frac{T_{a}\cdot 100}{T_{tot}}\;[\%]$$

Specific surface area *α* is calculated based on the total number of air voids *N* (Equation (2)).

(2) $$\alpha =\frac{4\cdot N}{T_{a}}\;[{mm}^{-1}]$$

The spacing factor *L* was calculated using one of two formulas, depending on the paste-to-air ratio *R* (Equations (3) and (4)).

(3) $$\mathrm{If}\;R>4.342,\;\mathrm{then}\;L=\frac{3\cdot \left[1.4\cdot {\left(1+R\right)}^{\frac{1}{3}}-1\right]}{\alpha }\;[mm]$$

(4) $$\mathrm{If}\;R\leq 4.342,\;\mathrm{then}\;L=\frac{P\cdot T_{tot}}{400*N}\;\left[mm\right]$$

The micropore content *A*_300_ is estimated based on the total length of the measurement line and the distribution of chord lengths divided into 28 size classes within the range of 0–4000 µm, with a defined size interval for each class.

The 2D method analysis was conducted according to guidelines described in the authors’ previous work [10], utilizing the Schwartz–Saltykov computational approach. At this research stage, the entire image surface (80 × 50 mm) was analyzed. The air content is calculated based on the area of air voids (Σ*Area*) and total measured area (*AOM*—Area of Measurement) (Equation (5)).

(5) $$A=\frac{\Sigma Area}{AOM}$$

Specific surface area *α* calculated based on the equivalent diameter of pores *EqD* (Equation (6)).

(6) $$\alpha_{1}=\frac{4}{3}\cdot \pi \cdot \frac{\Sigma EqD}{\Sigma Area}\;\left[{mm}^{-1}\right],$$

The spacing factor *L* was determined using one of two equations, depending on the value of the ratio *R* (Equations (7) and (8)).

(7) $$\mathrm{If}\;R>4.342,\;\mathrm{then}\;L=\frac{3*\left[1.4\cdot {\left(1+R\right)}^{\frac{1}{3}}-1\right]}{\alpha }\;[mm]$$

(8) $$\mathrm{If}\;R\leq 4.342,\;\mathrm{then}\;L=R\cdot \alpha \;\left[mm\right]$$

Micropore content is estimated using stereological principles and the classification of pores into 15 size classes, each with a width of 20 µm.

### 3.1. Results of Air Void Structure Parameters Calculated Using the 1D Method

**Figure 7:**
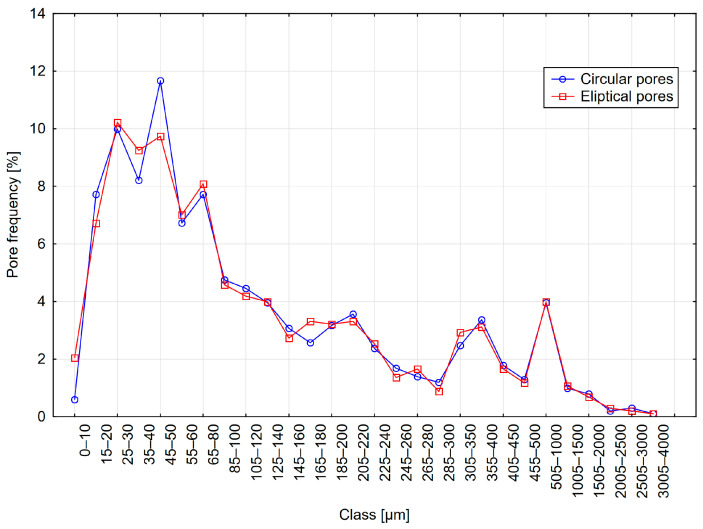
Distribution A—frequency of circular and elliptical pores using the 1D method.

**Figure 8:**
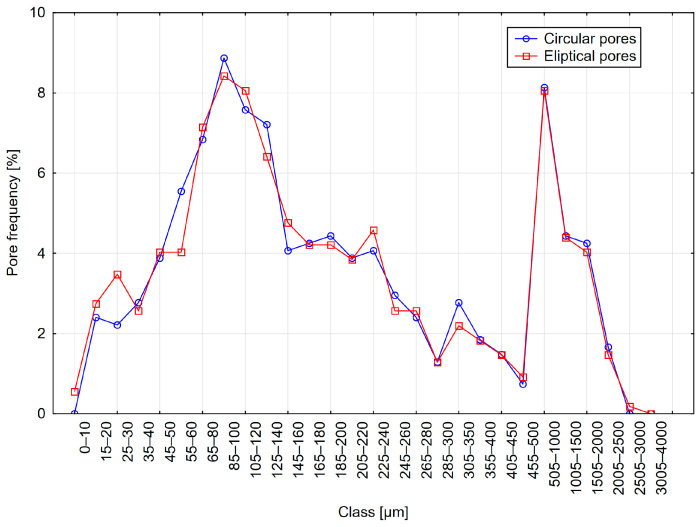
Distribution B—frequency of circular and elliptical pores using the 1D method.

**Figure 9:**
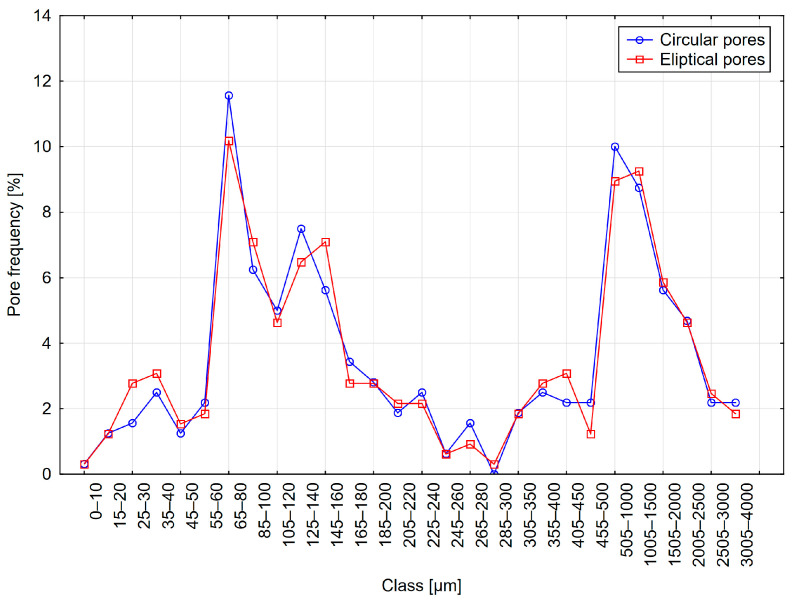
Distribution C—frequency of circular and elliptical pores using the 1D method.

The results are presented in Table 3 and Figure 7, Figure 8 and Figure 9. The table shows the results obtained for models with circular pores (labeled as Cr) and elliptical pores (labeled as El).

From the analysis of the results presented in Table 3, it can be observed that individual parameters differed slightly between variants with circular and elliptical pores. Similarly, when analyzing pore distribution graphs, the influence of pore shape on measurement results appears minor. However, it should be noted that in actual concrete, the influence of pore shape might be more significant. A considerable proportion of chords greater than 500 µm was recorded, amounting to approximately 10%, significantly affecting the calculations of micropore content. In contrast, the pore distributions selected for the models had a considerably lower content of such pores—around 2%. This discrepancy may result from the relatively small number of chords recorded during measurement.

### 3.2. Results of Air Void Structure Parameters Calculated Using the 2D Method

**Figure 10:**
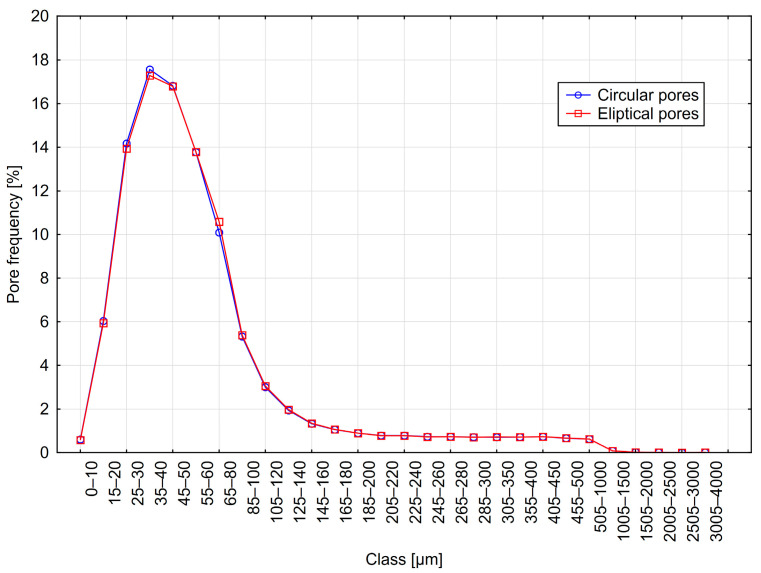
Distribution A—frequency of circular and elliptical pores using the 2D method.

**Figure 11:**
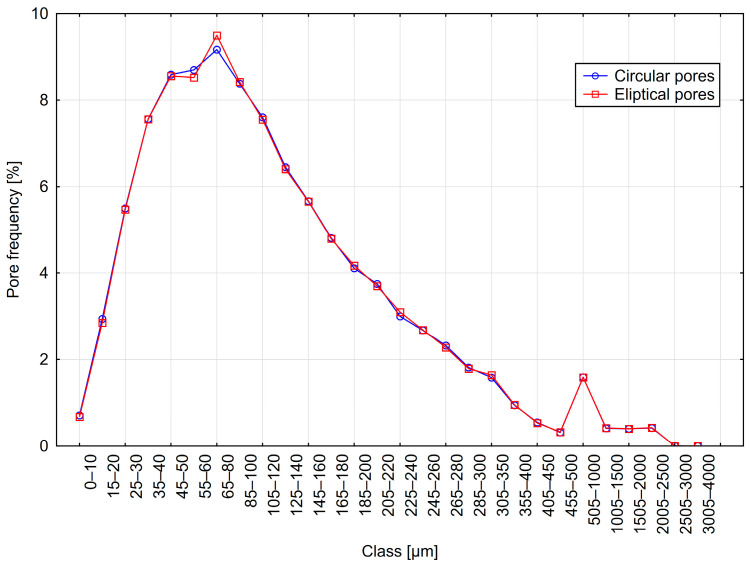
Distribution B—frequency of circular and elliptical pores using the 2D method.

**Figure 12:**
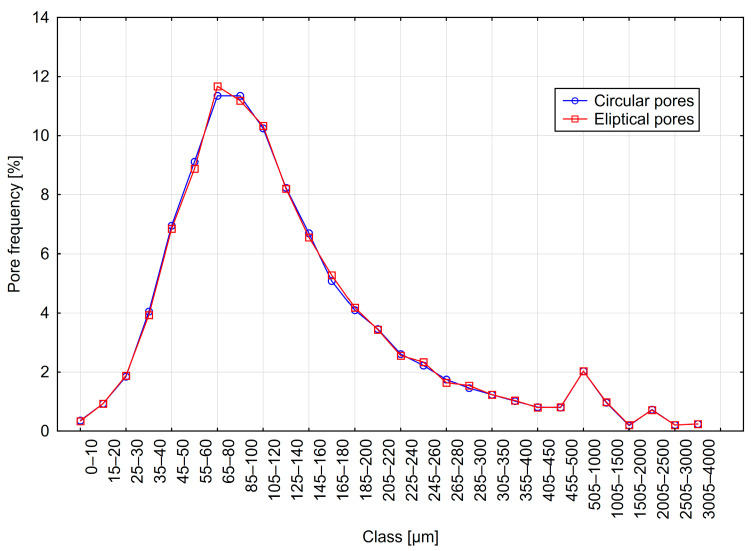
Distribution C—frequency of circular and elliptical pores using the 2D method.

The results are presented in Table 4 and Figure 10, Figure 11 and Figure 12. The table summarizes the results obtained for models with circular pores (labeled as Cr) and elliptical pores (as El).

Analyzing the results obtained using the 2D method, it can be observed that parameter values were nearly identical for variants with circular and elliptical pores. The pore distributions closely resemble those used to generate the models. Additionally, only minor differences are visible between distributions obtained from variants with circular and elliptical pores.

### 3.3. Comparison of Results Obtained by the 2D and 1D Methods

The agreement between results obtained using the 2D and 1D methods was evaluated using the indicator *v_k_* (Equation (9)), representing the root mean square relative deviation. The lower the *v_k_* value, the better the agreement between the compared results. The obtained results are presented in Table 5.

(9) $$\nu_{k}=100*\sqrt{\frac{\Sigma {\left(\frac{Y\;-\;y}{Y}\right)}^{2}}{n-1}}$$

where: (i) *Y*—values of individual parameters calculated using the 1D method, (ii) *y*—values of individual parameters calculated using the 2D method.

The analysis demonstrated a very strong agreement of the obtained results. The exception was the *A*_300_ parameter, with an agreement level of 18–21%, which can be considered sufficient. Based on the comparative analysis of the results, it was determined that pore shape has a secondary significance. Therefore, only circular pores were selected for comparison analysis. A comparison of 1D and 2D calculations on geometric models confirmed the authors’ previous findings [10].

### 3.4. Analysis of the Number, Size, and Resolution of Measurement Frames

**Figure 13:**
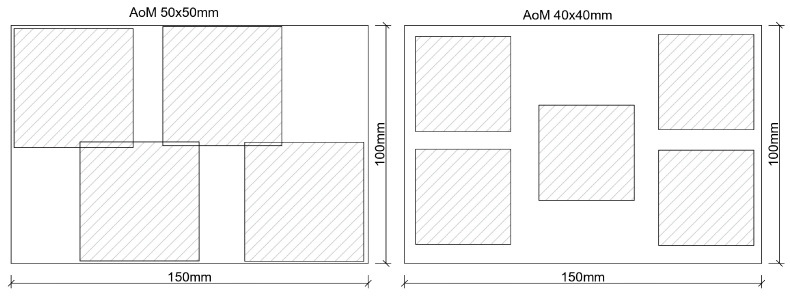
Arrangement of measurement frames on the surface of a sample with dimensions 150 × 100 mm.

The next stage of the research involved determining the required number and size of measurement frames necessary to obtain reliable results using the 2D method. Similarly to the approach presented previously, nine variants were adopted, including three levels of air content (3%, 5%, 8%) and three types of pore distribution (A, B, C). The study was conducted on 90 numerical models measuring 100 × 150 mm, which served as references. Measurements were carried out on these images, and air void structure parameters were calculated using the 1D and 2D methods. Subsequently, measurement frames with dimensions of 40 × 40 mm and 50 × 50 mm were extracted from the reference images. Generating larger high-resolution frames may result in file sizes exceeding 200 MB, which poses a significant challenge for image processing. Six variants for the number of frames were planned: (i) 1 × 40 × 40 (50 × 50) mm, (ii) 2 × 40 × 40 (50 × 50) mm, (iii) 3 × 40 × 40 (50 × 50) mm, (iv) 4 × 40 × 40 (50 × 50) mm, (v) 5 × 40 × 40 (50 × 50) mm, and (vi) 6 × 40 × 40 (50 × 50) mm. An example illustrating the method of extracting measurement frames is presented in Figure 13.

**Figure 14:**
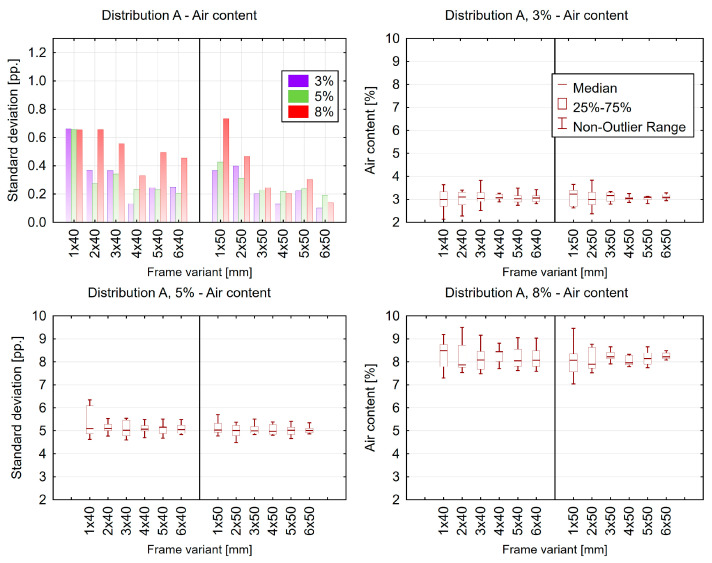
Example box plots illustrating the dispersion of air content results.

Measurements and calculations using the 2D method were performed on the extracted measurement frames, and the obtained results were compared with those from the reference images. Due to the large number of generated graphs, only an example for one selected variant is presented (Figure 14).

It was found that measurements and calculations performed on 50 × 50 mm frames exhibited better agreement in the obtained results. The smaller the frame area, the greater the impact of pores exceeding 500 µm in diameter on the results. Increasing the number of measurement frames improves result accuracy but also increases the labor intensity of the research. At this stage of the study, it can be assumed that obtaining satisfactory results requires at least three measurement frames of 50 × 50 mm or four frames of 40 × 40 mm, beyond which additional data provided minimal benefit.

**Figure 15:**
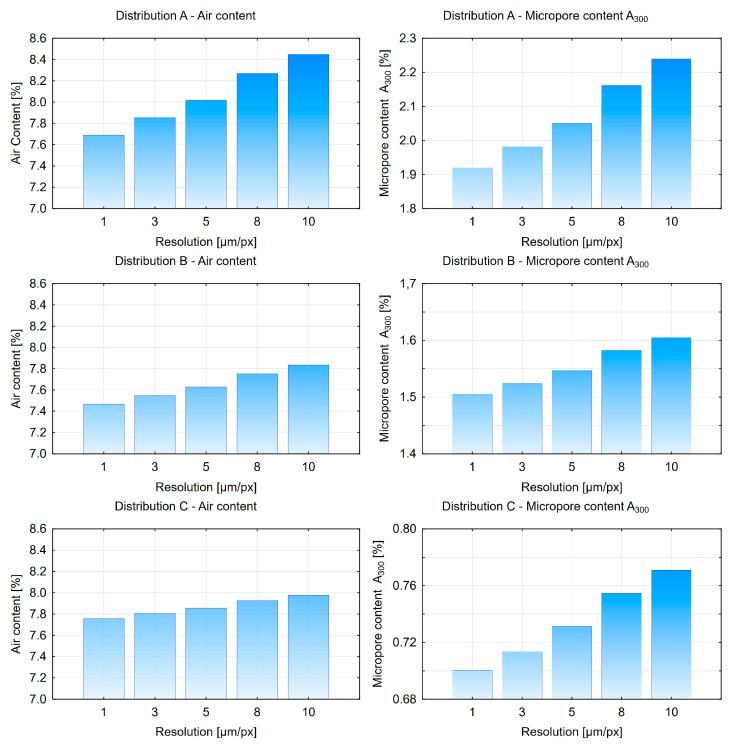
Air content and *A*_300_ micropore charts depending on image resolution.

**Figure 16:**
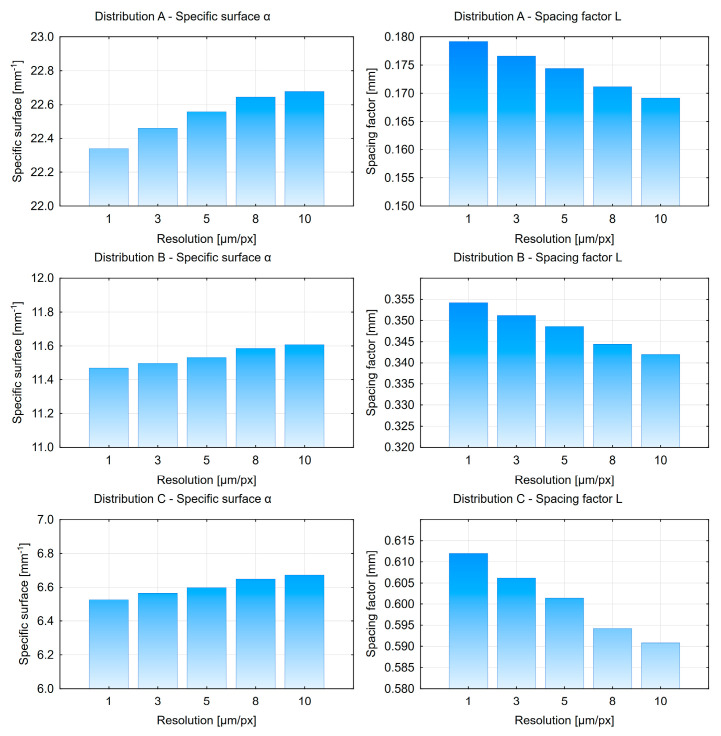
Specific surface area and spacing factor charts depending on image resolution.

The final stage of the research was the evaluation of the impact of image resolution on the calculation results. For each of the three pore distribution variants (A = 5%), five different images (80 × 50 mm) were generated with resolutions of 1, 3, 5, 8, and 10 µm/px. The results are presented in Figure 15 and Figure 16.

Based on the obtained results, it can be observed that the air content (*A*), specific surface area of pores (α), and micropore content (*A*_300_) increase as the image resolution decreases. The spacing factor *L* gradually decreases. This may result in an underestimation of the spacing factor value. This trend is associated with the progressive loss of pore shape fidelity at lower resolutions. It is anticipated that this effect would be more pronounced in real concrete samples. The analysis of a pore consisting of 3 pixels at a resolution of 3 µm/px results in a measured pore diameter of 9 µm (3 px × 3 µm/px = 9 µm). Consequently, a lower resolution would prevent the measurement of all pores with diameters smaller than 10 µm. It has been determined that the optimal solution is to use a resolution of 3 µm/px or higher. Therefore, it is recommended that a minimum resolution of 3 µm/px be used to ensure reliable measurements.

## 4. Conclusions

The study aimed to: (i) assess the impact of air void shape on computed parameters, (ii) compare results from 1D and 2D methods, and (iii) determine the optimal number, size, and resolution of image frames for reliable and repeatable analysis. The approach was based on geometrical air void models derived from measurements in pavement concretes.

The key finding of the analysis is that the use of a resolution of 3 µm/px or greater is a fundamental requirement for obtaining satisfactory results. Using larger pixel sizes results in a decrease in the spacing factor *L*, while other parameters tend to increase. Thus, lower resolution yields overly “optimistic” results, which are clearly misleading. Another critical requirement is to perform measurements over a sufficiently large area. However, due to technical constraints, the size of the measurement windows must be limited. In this study, the file size was restricted to a maximum of 200 MB. At the resolution of 3 µm/px, 50 × 50 mm frames offer an optimal balance between accuracy and file size. The study indicated that at least three such frames are needed, or more smaller ones. Given that EN 480-11 mandates testing on two samples, it is reasonable to assume that two measurement frames per sample (four in total) are required.

The comparative analysis of the results indicated that both geometric models and real concrete specimens yielded consistent values for the spacing factor *L* and the specific surface area *α*. While high-resolution computed tomography holds promise for comprehensive comparisons of 1D, 2D, and 3D methods, current resolution limitations constrain the size of analyzable specimens.

The study of air void shape showed that in the 2D method, pore shape had minimal impact on the results due to the use of equivalent diameter, which reduces sensitivity to geometry. Moreover, the higher number of detected voids in 2D further diminishes shape influence. In contrast, the limited number of measured chords in the 1D method means that irregularly shaped pores can significantly affect the outcome.

However, as it is necessary to verify the results on real concrete, further research will be conducted on real concrete specimens. It is also necessary to resolve issues related to the accurate separation of connected air voids.

## Figures and Tables

**Table 1:** Selected scanning methods and image resolution in various scientific studies.

	Author	Method	Resolution [µm/px]	Size [cm]
1D	Konkol [18]	Stereoscopic microscope Flatbed scanner	2.76 6.36	6.32 × 3.25
	Song [7]	Flatbed scanner	5.3	6.35 × 6.35
1D/2D	Murotani [9]	Flatbed scanner	30	6 × 6
	Wawrzeńczyk [10,16]	Stereoscopic microscope	6.75 1.13	8.25 × 6.19
2D	Arnesh Das [12]	Flatbed scanner	5.3	3 × 5 × 5
	Fonseca [11]	Flatbed scanner	7.94	4 × 4
3D	Kim [19]	Computer tomography	200	40.96 × 40.96
	Du Plessis [20]	Computer tomography	0.05	0.015

**Table 2:** Characteristics of air void structure parameters in 292 pavement concretes [16].

	P %	*T_tot_* mm	*N*	*A* %	*A*_300_ %	*α* mm^−1^	*L* mm
Mean	28.07	2522.56	1016	5.66	2.73	29.37	0.17
Median	28.50	2537.74	937	5.59	2.55	27.19	0.17
Sd	1.94	47.74	313	1.65	0.92	7.49	0.04
Min	21.00	2438.62	251	0.95	0.61	15.80	0.08
Max	35.50	2643.62	2221	11.83	6.04	55.80	0.33

P—volume of cement paste, *T_tot_*—total traverse length, *N*—number of chords, *A*—air content, *A*_300_—micropore content, *α*—specific surface, *L*—spacing factor, Sd—standard deviation, Min—minimum value, Max—maximum value.

**Table 3:** Results of air void structure parameters calculated using the 1D method.

1D	*A* %	*A*_300_ %	*α* mm^−1^	*L* mm	*N*
Distribution A					
A3_Cr	2.70	1.00	23.77	0.271	186
A3_El	2.74	1.01	23.86	0.268	193
A5_Cr	4.96	1.51	21.97	0.223	339
A5_El	4.97	1.56	22.16	0.221	339
A8_Cr	7.27	2.05	22.97	0.168	486
A8_El	7.29	2.05	23.22	0.165	495
Distribution B					
B3_Cr	3.14	0.70	10.88	0.55	109
B3_El	3.17	0.76	10.93	0.55	109
B5_Cr	4.97	1.21	11.87	0.41	171
B5_El	4.96	1.25	11.96	0.41	173
B8_Cr	7.45	1.84	11.68	0.32	261
B8_El	7.42	1.90	11.97	0.32	264
Distribution C					
C3_Cr	3.37	0.30	6.18	0.94	65
C3_El	3.31	0.32	6.24	0.94	65
C5_Cr	4.94	0.60	6.55	0.75	102
C5_El	4.90	0.53	6.70	0.74	103
C8_Cr	7.74	0.63	6.00	0.60	153
C8_El	7.79	0.64	6.24	0.58	156

*A*—air content, *A*_300_—micropore content, *α*—specific surface, *L*—spacing factor, *N*—number of chords.

**Table 4:** Results of air void structure parameters calculated using the 2D method.

2D	*A* %	*A*_300_ %	*α*_1_ mm^−1^	*L*_1_ mm	*N*
Distribution A					
A3_Cr	3.07	0.78	22.35	0.27	8286
A3_El	3.07	0.78	22.36	0.27	8282
A5_Cr	5.03	1.27	22.31	0.22	13656
A5_El	5.04	1.27	22.34	0.22	13651
A8_Cr	7.77	1.94	22.31	0.18	20980
A8_El	7.78	1.94	22.33	0.18	20973
Distribution B					
B3_Cr	2.99	0.61	11.48	0.54	2216
B3_El	2.99	0.61	11.50	0.54	2218
B5_Cr	4.98	0.98	11.27	0.43	3622
B5_El	4.98	0.98	11.29	0.43	3623
B8_Cr	7.94	1.52	11.08	0.36	5547
B8_El	7.94	1.52	11.10	0.36	5545
Distribution C					
C3_Cr	3.09	0.28	6.60	0.92	1022
C3_El	3.09	0.28	6.61	0.92	1021
C5_Cr	5.03	0.51	6.99	0.70	1961
C5_El	5.03	0.51	6.99	0.70	1964
C8_Cr	8.02	0.76	6.67	0.59	2920
C8_El	8.03	0.75	6.66	0.59	2917

*A*—air content, *A*_300_—micropore content, *α*_1_—specific surface, *L*_1_—spacing factor, *N*—number of air voids.

**Table 5:** Agreement between the results obtained from the standard and surface methods.

*v_k_*				
	*A* %	*A*_300_ %	*α*_1_ mm^−1^	*L*_1_ mm
Distribution A	10.89	19.60	4.82	4.90
Distribution B	5.75	20.53	6.38	8.68
Distribution C	6.54	18.44	10.37	5.56

*A*—air content, *A*_300_—micropore content, *α*_1_—specific surface, *L*_1_—spacing factor.

## Data Availability

The original contributions presented in this study are included in the article. Further inquiries can be directed to the corresponding author(s).
